# Roadmap for Postnatal Brain Maturation: Changes in Gray and White Matter Composition during Development Measured by Fourier Transformed Infrared Microspectroscopy

**DOI:** 10.1021/acschemneuro.3c00237

**Published:** 2023-08-04

**Authors:** Marta Peris, Núria Benseny-Cases, Gemma Manich, Oriana Zerpa, Beatriz Almolda, Àlex Perálvarez-Marín, Berta González, Bernardo Castellano

**Affiliations:** † Department of Cell Biology, Physiology and Immunology, Institute of Neuroscience, Universitat Autònoma de Barcelona, Bellaterra, 08193 Barcelona, Spain; ‡ Biophysics Unit. Department of Biochemistry and Molecular Biology, Universitat Autònoma de Barcelona, Bellaterra, 08193 Barcelona, Spain; § Department of Morphological Sciences, Universitat Autònoma de Barcelona, Bellaterra, 08193 Barcelona, Spain

**Keywords:** myelination, development, white matter, gray matter, μFTIR

## Abstract

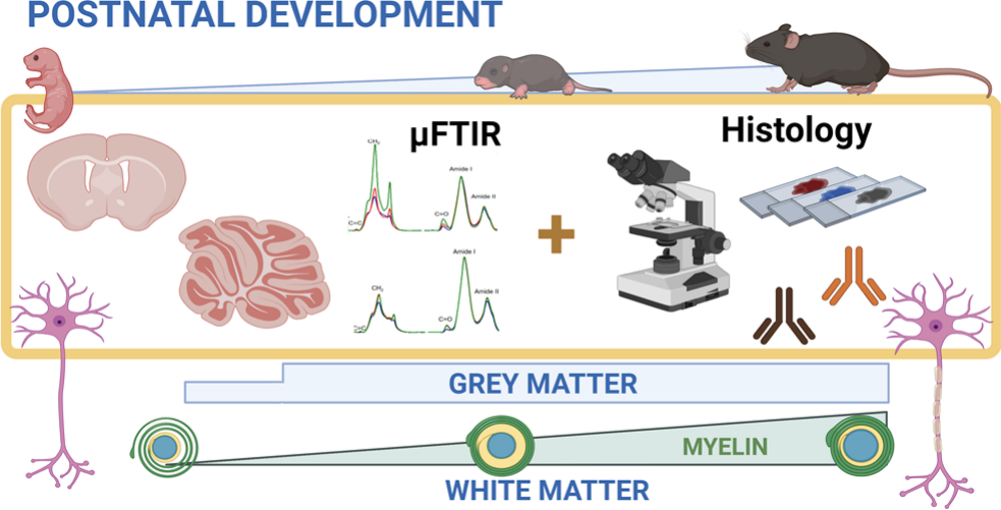

Key events in postnatal brain development, such as neuronal migration, synaptogenesis, and myelination, shape the adult brain. These events are reflected in changes in gray and white matter (GM and WM) occurring during this period. Therefore, precise knowledge of GM and WM composition in perinatal brain development is crucial to characterizing brain formation as well as the neurodevelopmental disruption observed in diseases such as autism and schizophrenia. In this study, we combined histochemical and immunohistochemical staining with biochemical and biophysical analyses using Fourier transform infrared (IR) microspectroscopy (μFTIR) to better understand the chemical changes during postnatal developmental myelination. For this purpose, we analyzed the GM and WM in the mouse brain and cerebellum (strain C57BL/6) from postnatal day 0 (P0) to day P28 and established presumed correlations between staining and IR data. IR spectra allowed the (i) quantification of lipid and protein content through the CH_2_/amide I ratio, (ii) determination of chemical characteristics of lipids, such as the presence of unsaturated bonds in the carbonate chain or carbonyls from ester groups in the polar head, and (iii) determination of the protein secondary structure (α-helix and intramolecular β-sheets). The results indicate that the increase in the CH_2_/amide I ratio calculated from the μFTIR data correlates well with lipid histochemical staining. IR data indicated a change in the lipid composition in WM since carbonyl and unsaturated olefinic groups do not increase when lipids accumulate during myelination. Our correlation analysis between IR data and immunohistochemical staining of myelin-associated proteins revealed that myelin oligodendrocyte protein correlated well with lipid accumulation, while myelin basic protein appeared before lipid modifications, which indicated that myelin-associated proteins and lipid deposition were not synchronic. These events were related to a decrease in the intramolecular β/α protein ratio. Our results indicate that lipids and proteins in WM substantially change their composition due to primary myelination, and according to results obtained from staining, these modifications are better described by lipid histochemical staining than by immunohistochemistry against myelin-related proteins. In conclusion, μFTIR can be a useful technique to study WM during perinatal development and provide detailed information about alterations in the chemical composition related to neurodevelopmental diseases.

## 1 Introduction

Key processes of central nervous system (CNS) maturation occur during perinatal development, including oligodendrogenesis, primary myelination, and neural network formation through synaptogenesis and synaptic pruning.^1,2^ Alterations in these processes can lead to several neurodevelopmental diseases, such as autism and hypomyelination.^3,4^ Postnatal brain maturation under physiological conditions is reflected in modifications of the gray matter (GM) and white matter (WM) composition, and their alteration can be related to pathophysiological mechanisms involved in neurodevelopmental diseases.

Determination of the lipid and protein composition of GM and WM in experimental animal models, such as mice and rabbits, has been obtained by chromatography, mass spectrometry, or imaging techniques in whole-brain samples and isolated myelin,^5–9^ techniques that lack area specificity and have few time points of study. In humans, these changes have been described using imaging and chromatography.^10–15^ Primary myelination in WM has been extensively studied using histochemical techniques, such as Luxol Fast Blue (LFB), Sudan Black (SB), and Oil Red O (ORO) staining, and immunohistochemistry against myelin sheath-specific proteins, such as myelin basic protein (MBP), proteolipid protein (PLP), and myelin oligodendrocyte glycoprotein (MOG), in animal brains.^16^ However, histochemical techniques provide only partial molecular information and do not describe detailed biochemical modifications in the GM and WM.

Current knowledge of postnatal brain composition is limited to the description of general changes. In mice, during the first early phase of 10 days, changes are related to brain growth since the total protein content, phospholipids, cholesterol, and nucleic acids increase globally. In the second phase, from day 14 onward, the brain composition is characterized by abrupt increases of cerebrosides and sulfatides due to myelin formation, which lasts for 8 days and decreases onward.^17^ Specific investigation of myelin-enriched fractions reveals a predominance of phosphatidylcholine at postnatal day (P) 15, followed by an increase in monounsaturated fatty acids and phosphatidylserine, phosphatidylinositol, and phosphatidylethanolamine because of myelin formation, as similarly observed in rats.^5,9^ Therefore, a general characterization of GM and WM changes during postnatal development with complete and specific biochemical and molecular features, spatial resolution, and representative points of study of postnatal development is needed.

One of the most sensitive techniques for analyzing the biochemical composition of biological samples, including fixed and nonfixed tissue slices, is Fourier transform infrared microspectroscopy (μFTIR).^18^ μFTIR provides complete information on the functional groups of lipids and protein secondary structures, while sample handling prior to analysis is minimized. μFTIR has been used in neuroscience as an interesting tool for studying tissues under homeostatic conditions, such as WM and GM in adult mice and humans during aging,^19–21^ or to study neurodegenerative diseases.^22–26^ In the case of postnatal development, only one study determined changes in the hippocampal formation by μFTIR in 6, 30, and 60 day-old rats.^27^

The technical advantages of μFTIR and its proven efficacy in CNS tissues make it a suitable option for studying changes in brain composition, especially lipids, during perinatal development. Owing to their key role in proper and abnormal brain maturation, data on specific biochemical changes in GM and WM can provide a deeper insight into relevant molecular information, especially when comparing μFTIR results with standard myelin histochemical and immunohistochemical methods. Therefore, this study sets the basis for understanding GM and WM composition during mammal brain maturation, as well as for studying its alterations in animal models of neurodevelopmental diseases and in the developing human brain.

## 2 Results

### 2.1 μFTIR Data Show Important Modifications in WM Composition during Postnatal Development

To study the changes in the general composition of GM and WM during postnatal development, we analyzed the spectra of the following CNS areas: for GM, we studied the cerebral cortex and cerebellar molecular (GM-M) and granular layers (GM-G); for WM, we investigated the corpus callosum (CC) and cerebellar arbor vitae.

**Figure 1:**
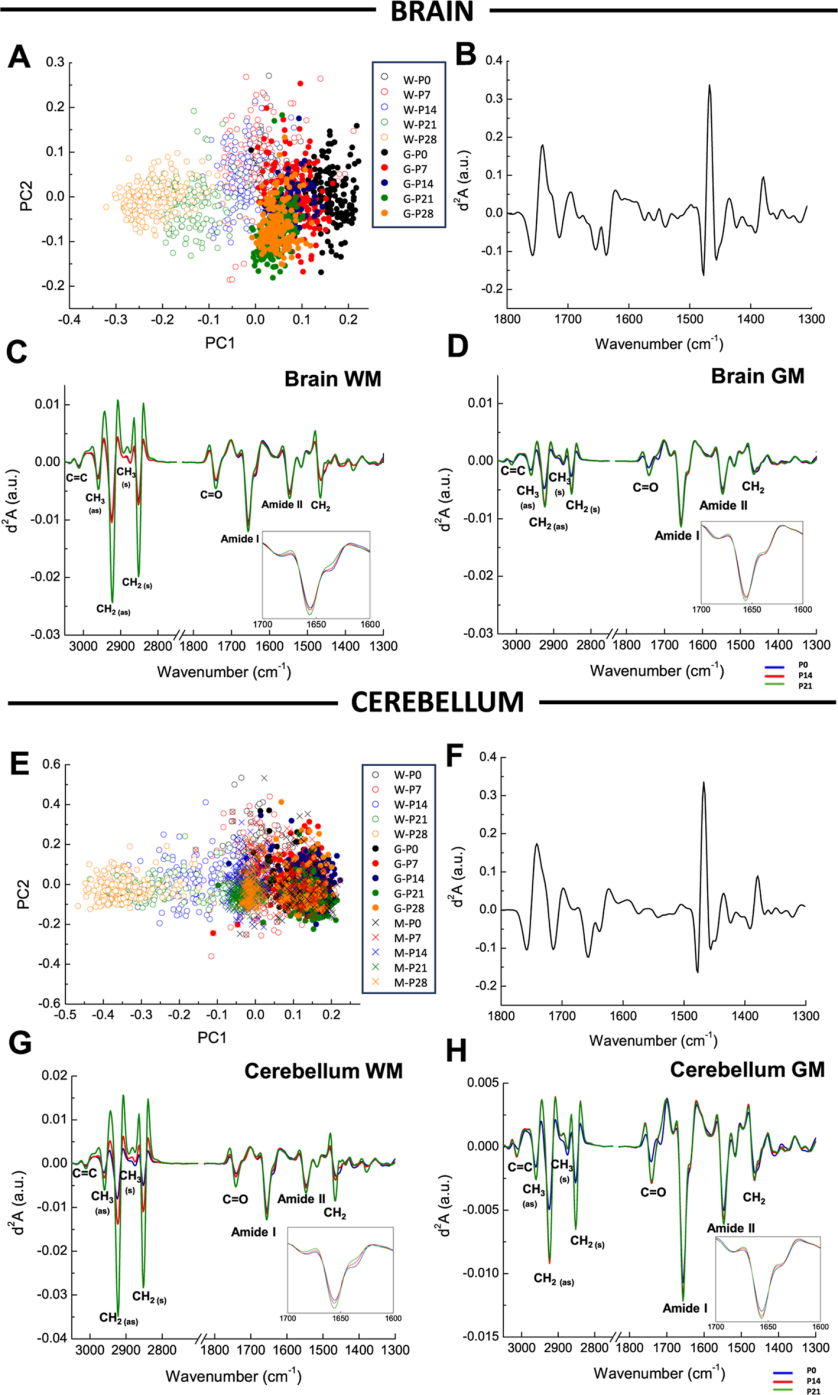
PCA in the second derivative spectra of μFTIR from brain and cerebellum WM and GM during postnatal development. Score plots (A,E) and loading plots (B,F) of PCA in the fingerprint region of the brain and cerebellum GM and WM spectra are shown. Clear differences are observed in GM (full circles, G) and WM (empty circles, W) of the brain, and GM in the granular (GM-G) and molecular (GM-M) layers (full circles (G) and crosses (M), respectively) and WM (empty circles, W). A normalized average of the second derivative spectra in WM and GM areas of the mouse brain (C,D) and the cerebellum (G,H) at P0 (blue lines), P14 (red lines), and P28 (green lines) is represented. Peaks corresponding to functional groups are illustrated in groups according to their absorbance (d^2^*A*, in arbitrary units) of the following bands (wavenumber in cm^–1^): 3012 cm^–1^ (C=CH, unsaturated olefinic group), 2921 cm^–1^ (CH_2_ asymmetric stretching vibrations), 1743 cm^–1^ (C=O, carbonyl group), 1656 cm^–1^ (α-helix secondary protein structure), 1637 cm^–1^ (β-sheet secondary protein structure).

To determine the specific bands that change during development, a principal component analysis of the fingerprint region (1800–1300 cm^–1^) was carried out. The score plot and loadings of the analysis of all brain and cerebellar spectra (GM and WM) are presented in Figure 1A,E and B,F, respectively. A separate analysis of GM and WM was also performed with similar loadings and score plots (data not shown).

For the analysis of the brain spectra, the score plot mainly separates the different areas and ages on PC1 (78% of discrimination). The PC1 loading plot shows the bands that distinguish the spectra of WM and GM during development in the fingerprint region. The bands in the PC1 correspond to carbonyl groups (band at 1740 cm^–1^), amide I (1656 cm^–1^ for α-helix structures and 1637 cm^–1^ for β-sheet structures), and CH groups (bands at 1470 and 1380 cm^–1^). Since principal component analysis (PCA) was performed on the second derivative of the spectra, the spectra on the positive axis of the score plot contained more proteins (negative band in the loading plot) and fewer lipids (positive band in the loading plot) than the spectra on the negative part of the score plot.

The score plot shows clear differences between GM and WM, especially at more advanced stages, showing a higher lipid content in WM than in GM. The analysis of WM and GM showed that mature GM composition was achieved at 7 days since only the P0 spectra differed from the rest of the GM data. However, for WM, P0, P7, and P14 showed similar spectra (the spectra colocalized in the score plot), whereas P21 and P28 differed from the early stages, showing that the WM composition was still changing until 21 and 28 days.

PCA for the cerebellum spectra for each area analyzed (GM-G, GM-M, and WM) is shown in panels E and F. The PC1 loading plot (43% of discrimination) shows very similar bands to those for the brain spectra. For GM-G, all spectra for all ages colocalized on the right part of the score plot, indicating that no changes could be detected in the fingerprint region using μFTIR. However, GM-M P0 and P7 spectra colocalized with GM-G at the positive part of the graph, while P14, P21, and P28 spectra were situated more in the center of the score plot, indicating a higher content of lipids at those stages. For cerebellar WM, as observed for brain data, a higher lipid content was detected because the spectra were found in the negative part of the score plot. In this case, the increase in lipid (shift of the spectra to the negative part) starts at P14, at an earlier stage than in the brain.

The changes detected in the PCA could also be observed in the second derivative (d^2^) of the average spectra for each condition, normalized by amide I, as shown in Figure 1C,D,G,H. Changes in CH could be observed more accurately in the 3050–2800 cm^–1^ region. The most pronounced changes in the IR spectra occurred in the CH_2_ band (d^2^*A*_2921_ and d^2^*A*_2852_). Changes in the C=O band (d^2^*A*_1743_) showed a marked gradual increase with development, especially in the cerebellar WM. In the cerebellar GM and brain, the modifications were almost imperceptible. This was also the case for the amide I band (d^2^*A*_1656_), corresponding to the α-helix secondary protein structure. However, bands corresponding to the β-sheet structure (d^2^*A*_1637_) and C=CH (d^2^*A*_3012_) showed no apparent differences between the groups as animals grew. The average spectra calculated from the spectra of each condition are also shown in Figure S1.

To statistically analyze the changes observed in PCA and in the average spectra, we first analyzed the changes in the lipid/protein ratio during development. For this, we used the intensity of the second derivative of the CH_2_ (d^2^*A*_2952_) band, representing the total lipid content, and amide I (d^2^*A*_1656+1637_) representing the protein absorption.^8,25^ Although the band intensity in the absorption spectrum would be proportional to the amount of lipid or protein, the efficiency in the IR absorption varies depending on the bond and the vibration mode; therefore, the CH_2_/amide I ratio (d^2^*A*_2952_/d^2^*A*_1656+1637_; CH_2_/(α + β)) used in this study cannot be directly interpreted as the actual lipid/protein ratio but as an estimation of the variation in the lipid/protein ratio.

**Figure 2:**
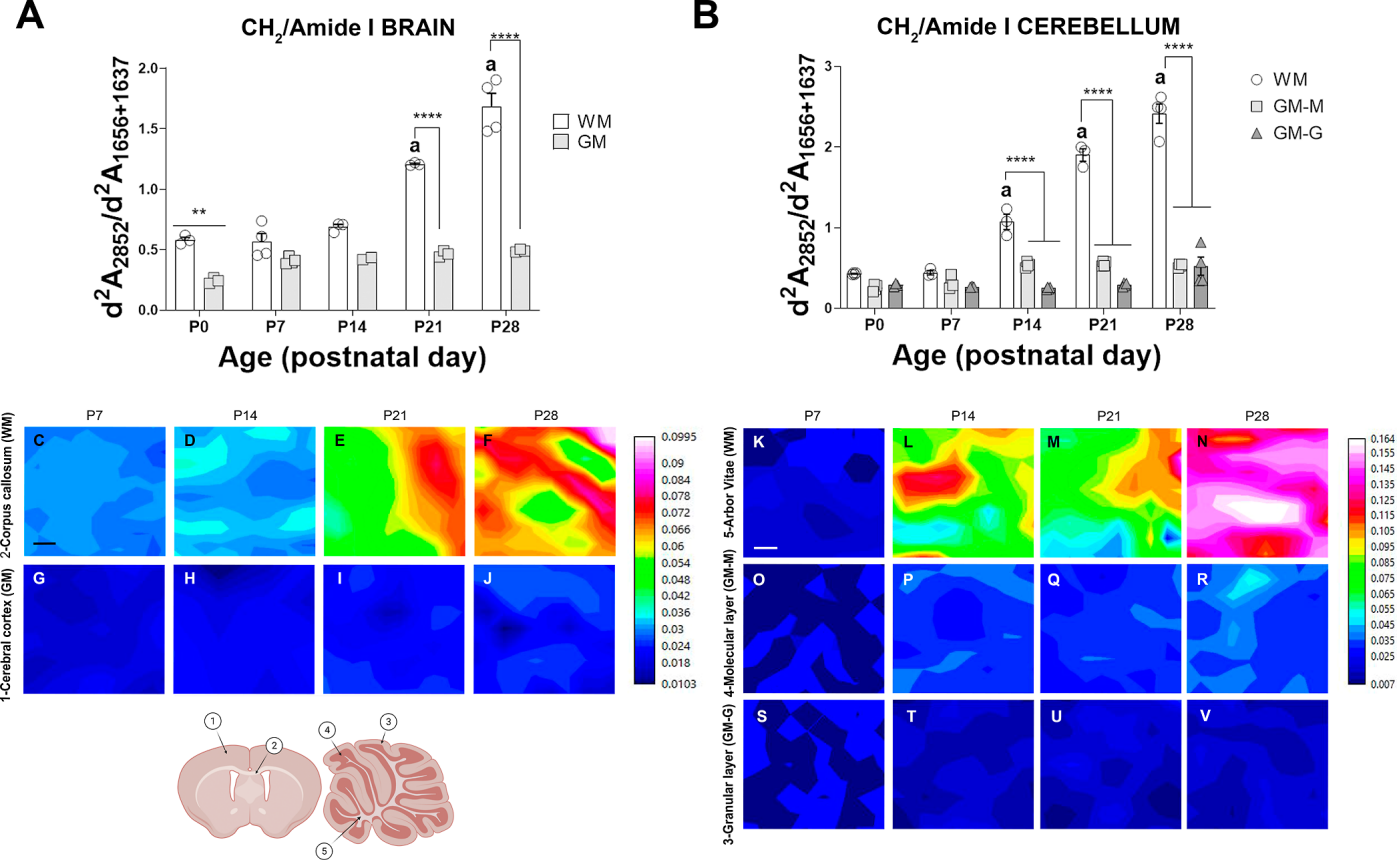
Changes in the CH_2_/amide I ratio in the second derivative spectra of μFTIR obtained from brain and cerebellum WM and GM during postnatal development. (A,B) Quantification of the CH_2_/amide I ratio (CH_2_/α + β) after second derivatives of the respective absorbances (d^2^*A*_2852_/d^2^*A*_1656+1637_) in the brain (A) and cerebellum (B) GM and WM areas during postnatal development. Data are represented as the mean ± standard error mean (SEM) Figure 6; Figure 6; Figure 6. Statistical treatment was performed with the two-way ANOVA test (time effect *p* < 0.0001; region effect *p* < 0.0001), and posthoc comparisons of differences between time points or areas were detected with Tukey’s posthoc test and represented as follows: **p < 0.01 and ****p < 0.0001 when WM compared to GM; “a” *p* < 0.0001 when compared to the previous time point. (C–V) Tissue heat maps represent the CH_2_/amide I (d^2^*A*_2852_/d^2^*A*_1656+1637_; CH_2_/α + β) ratio in the CC (C–F), cerebral cortex (G–J), arbor vitae (K–N), molecular layer (O–R), and granular layer (S–V) of the cerebellum. Scale bar = 10 μm. The bottom left is a representative drawing, where the studied areas are indicated: 1-cortex, 2-CC, 3-molecular layer, 4-granular layer, and 5-arbor vitae. Created with Biorender.com.

In the brain GM and granular cerebellum layers, no significant changes were observed in the CH_2_/amide I ratio throughout development, while in the molecular layer, the CH_2_/amide I ratio was significantly increased at postnatal day 14 (P14) (*p < 0.05, P0 GM-M vs P14 GM-M) and then remained stable (Figure 2B). In contrast, significant changes were observed in the WM during development. In the early stages, the CH_2_/amide I ratio in both the cerebellum and brain was slightly higher than that in the GM and remained stable until P7 and P14, respectively (Figure 2). After these time points, this ratio significantly increased, and lipids progressively accumulated in the WM at later time points. In general, WM areas showed changes that appeared later and were larger than those observed in GM areas (two-way ANOVA, region effect ****p < 0.0001 in the brain and cerebellum).

To eliminate the contribution of possible changes in sample thickness, we used CH_2_/amide I ratios; however, analysis of raw data for lipid (CH_2_) and amide I absorption, as shown in Supporting Information Figures S2 and S3, confirmed that changes observed in the CH_2_/amide I ratio corresponded to modifications in the total lipid content. Little change was observed in amide I absorption, while a significant increase was observed for the lipid band, corroborating the increase in lipids during development in WM.

### 2.2 Modifications in the Biochemical Composition of Lipids and Proteins Are Related to Primary Myelination

**Figure 3:**
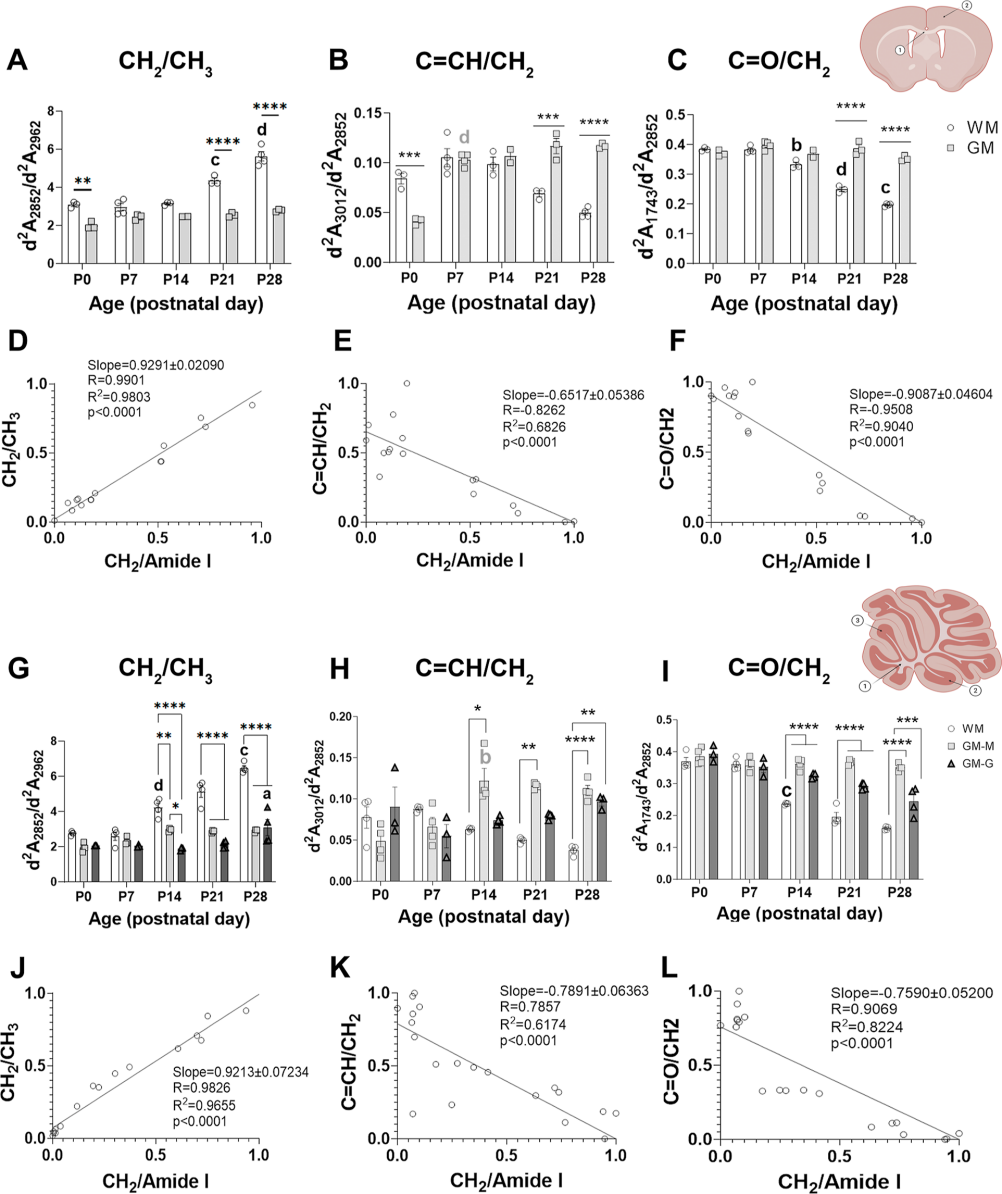
Biochemical composition of lipids in the brain and cerebellar WM and GM of WT mice during postnatal development. (A–C,G–I) Representation of the ratio of the second derivative of the absorbances (d^2^*A*) of CH_2_ (A,G), unsaturated olefinic (B,H)m and carbonyl (C,I) functional groups compared to the CH_3_ or the CH_2_/amide I ratio (an estimation of the total lipid content of the brain GM and WM (A,B) and cerebellar GM-formed by the molecular layer (GM-M) and granular layer (GM-G)- and WM from postnatal day 0 (P0) to P28. Data are represented as median ± SEM. Statistical treatment for brain areas was performed with a two-way ANOVA test (time effect ****p < 0.0001; region effect for the CH_2_ and unsaturated olefinic groups ****p < 0.0001 and for the carbonyl group *p* = 0.0003) and Tukey’s posthoc comparisons (*p < 0.05, **p < 0.01, ***p < 0.001, and ****p < 0.0001 WM compared to GM); “a” *p* < 0.05, “b” *p* < 0.01, “c” *p* < 0.001, and “d” *p* < 0.0001 when compared to the previous time point). Statistical treatment for cerebellar areas was performed with a two-way ANOVA (time effect n.s. for olefinic groups; *p* < 0.0001 for the CH_2_ and carbonyl groups; *p* < 0.0001 regional effect for all three groups) and Tukey’s posthoc comparisons (indicated as in the brain). (D–F,J–L) Correlation between the normalized CH_2_/amide I ratio (between 0 and 1) and the normalized ratio of CH_2_/CH_3_ ratio (D,J) unsaturated olefinic group (E,K) or carbonyl group (F,L) in WM. See Materials and Methods for further details about the obtention of normalized values. Drawings indicate areas of study: 1-CC, 2-cortex; 1-arbor vitae, 2-granular layer, and 3-molecular layer. Created with Biorender.com.

To study the changes in the biochemical composition of lipids in different brain and cerebellar areas during development, we measured the amounts of unsaturated olefinic (d^2^*A*_3012_; C=CH) and carbonyl (d^2^*A*_1743_; C=O) normalized to the total lipid content (d^2^*A*_2852_, CH_2_) (Figure 3). Because the absorption of the different groups is highly dependent on the efficiency of the IR absorption, changes in these ratios represent changes in composition but do not represent the exact ratio of these groups in the sample. Also, we studied changes in the ratio CH_2_/CH_3_ (d^2^*A*_2852_/d^2^*A*_2959_), which could be highly dependent on changes in the lipid/protein ratio.^25^

In the brain GM, carbonyl groups remained constant across all time points (Figure 3A–C), while CH_2_ and the unsaturated olefinic functional group showed lower levels compared to WM that were lost at P7. In brain WM, CH_2_ increased following a pattern similar to that described for the CH_2_/amide I ratio. Since lipids have a larger amount of CH_2_ than CH_3_ compared to proteins, which have a more similar amount of these two groups in their lateral chains, an increase in the CH_2_/amide I ratio will translate into an increase in the CH_2_/CH_3_ ratio. This is demonstrated in the correlation graph, which shows a strong correlation between these two groups (Figure 3D,J). Besides, in WM, carbonyl and olefinic unsaturated functional groups normalized by total lipid content decreased from P21 onward, thus presenting lower amounts than in GM (Figure 3A,B). Considering that, as we previously described, the total lipid content of WM increases during development, carbonyl and olefinic functional groups express a dynamic pattern opposite that of the CH_2_/amide I ratio.

In the case of cerebellar GM, the CH_2_ group levels remained constant (Figure 3G), but we found a significant increase in unsaturated olefinic bonds throughout postnatal development, first in the molecular layer at P14 (two-way ANOVA, posthoc Tukey’s P0–P14 ****p < 0.0001) and later in the granular layer at P28 (two-way ANOVA, posthoc Tukey’s P14–P28 ****p < 0.0001) (Figure 3G). Finally, carbonyl levels decreased only in the GM-G (Figure 3I; two-way ANOVA, posthoc Tukey’s P14–P28 ***p < 0.001). In the cerebellar WM (arbor vitae), CH_2_ followed a similar pattern to the one observed for the CH_2_/amide I ratio. Unsaturated and carbonyl groups progressively and significantly decreased (Figure 3H,I, two-way ANOVA, posthoc Tukey’s P0–P14 ****p < 0.0001 and P7–P28 *p < 0.05, respectively). Owing to the gradual growth of unsaturated olefinic groups in GM and the decrease of both functional groups in WM, significant differences in the content of both functional groups were found between GM and WM at later developmental stages (Figure 3H,I). As similarly described for the brain, in WM, unsaturated olefinic and carbonyl functional groups showed an expression dynamic pattern opposite that of the CH_2_/amide I ratio.

To better analyze the composition of lipids accumulating in WM during maturation, the correlation between the different ratios and the CH_2_/amide I ratio normalized between 0 and 1 was calculated using linear regression. A moderate-to-poor linear correlation was observed for the unsaturated olefinic groups (Figure 3E,K) while lipids accumulate. However, a strong linear correlation of carbonyl groups with the CH_2_/amide I ratio is shown, which demonstrates a dependent decrease in the ratio of carbonyl groups (Figure 3F). In the WM of the cerebellum, the correlation is poor for olefinic unsaturation (Figure 3L), as observed in the brain, while the carbonyl functional group showed a strong and linear decrease that correlated with an increase in the lipid content (Figure 3K). Therefore, in both cases, the increase in lipids was related to a decrease in both functional groups normalized by the total lipid (Figure 3E,F,K,L), and the correlation was especially strong for carbonyl groups in both the brain and cerebellum (Figure 3F,L), indicating that myelin lipids synthesized during primary myelination are poor in carbonyl groups and olefinic unsaturation.

**Figure 4:**
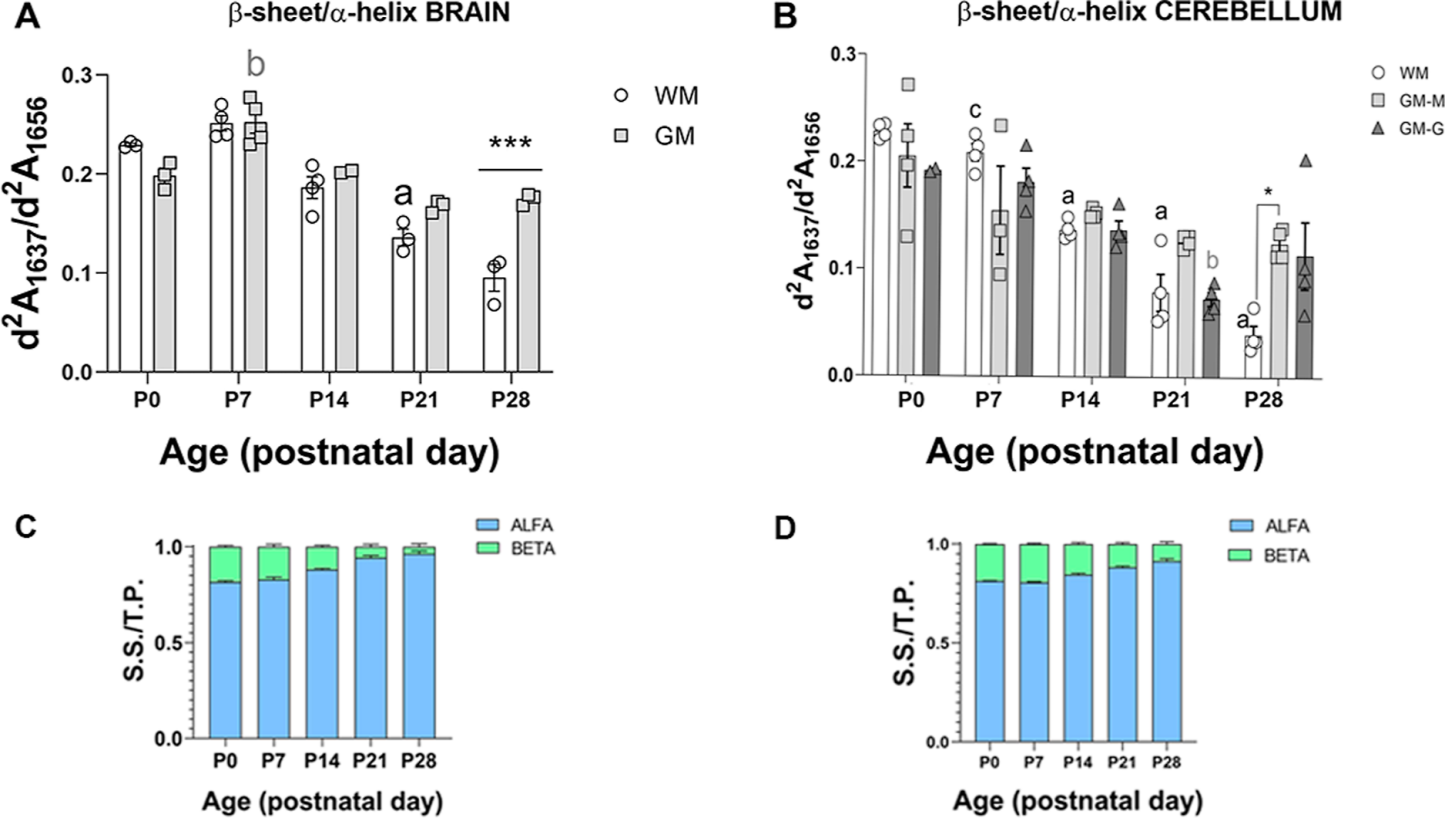
Secondary structure of proteins in the cerebellum and brain WM and GM of WT mice during postnatal development. (A,B) Representation of the ratio of the second derivative of the absorbances (d^2^*A*) of amide I (β-sheet)/amide I (α-helix) of proteins (d^2^*A*_1637_/d^2^*A*_1656_) in the (A) cerebral cortex (brain GM) and CC (WM), CC in the (B) cerebellar WM, molecular layer, and granular layer of GM from postnatal day 0 (P0) to P28. Data are represented as median ± SEM. Statistical treatment was performed with the two-way ANOVA test (for the brain: time effect *p* < 0.0001, region effect *p* = 0.003; for the cerebellum: time effect *p* < 0.0001; region effect n.s.), and Tukey’s posthoc comparisons (*p < 0.05 and ***p < 0.001, WM compared to GM; “a” *p* < 0.05 and “b” *p* < 0.01 compared to a previous time point). (C,D) α-helix (blue) and β-sheet (green) secondary structure proportions out of total protein (measured as amide I) in brain and cerebellar WM during postnatal development. S.S. = secondary structure (α-helix, or β-sheet) and T.P. = total protein (amide I).

The amide I is sensitive to the protein’s secondary structure, so the ratio d^2^*A*_1637_/d^2^*A*_1656_ (β-sheet/α-helix) was used to study changes in the secondary structure of proteins during development. In the brain GM, the β-sheet/α-helix ratio slightly increased at P7 and then remained stable across all time points (Figure 4A). In the GM of the cerebellum, no changes were observed in the granular layer, whereas in the molecular layer, the β-sheet/α-helix ratio decreased from P7 to P21 (two-way ANOVA, posthoc Tukey P7–P21 GM-M **p < 0.01) and then returned to previous levels at P28 (Figure 4B).

A progressive decrease in the β-sheet/α-helix ratio was observed in the WM of both the brain and cerebellum. In the brain and cerebellar WM, the β-sheet/α-helix ratio decreased from P7 onward (two-way ANOVA, posthoc Tukey P7–P21 cerebellar WM ***p = 0.0001; Figure 4A,B); at P28, in the brain and cerebellar WM, the β-sheet/α-helix ratio was significantly decreased compared to GM (Figure 4A,B). Analysis of the proportion of α-helix or β-sheet compared to total protein (amide I) in WM of both the brain and cerebellum (Figure 4C,D) suggests that the α-helix secondary structure is by far the most dominant during all time points and, coinciding with myelination, it continues to increase throughout development.

### 2.3 Changes in the Brain Composition Associated with Primary Myelination Are Well Reflected by LFB, SB and ORO Histochemical Staining

Our data indicate that the most important changes in brain composition during postnatal development are related to WM and primary myelination. Myelination has traditionally been studied using other available experimental histological techniques. Therefore, to study how changes in postnatal brain composition are reflected by these techniques, we compared and correlated the μFTIR results with those obtained using histochemistry or immunohistochemistry.

**Figure 5:**
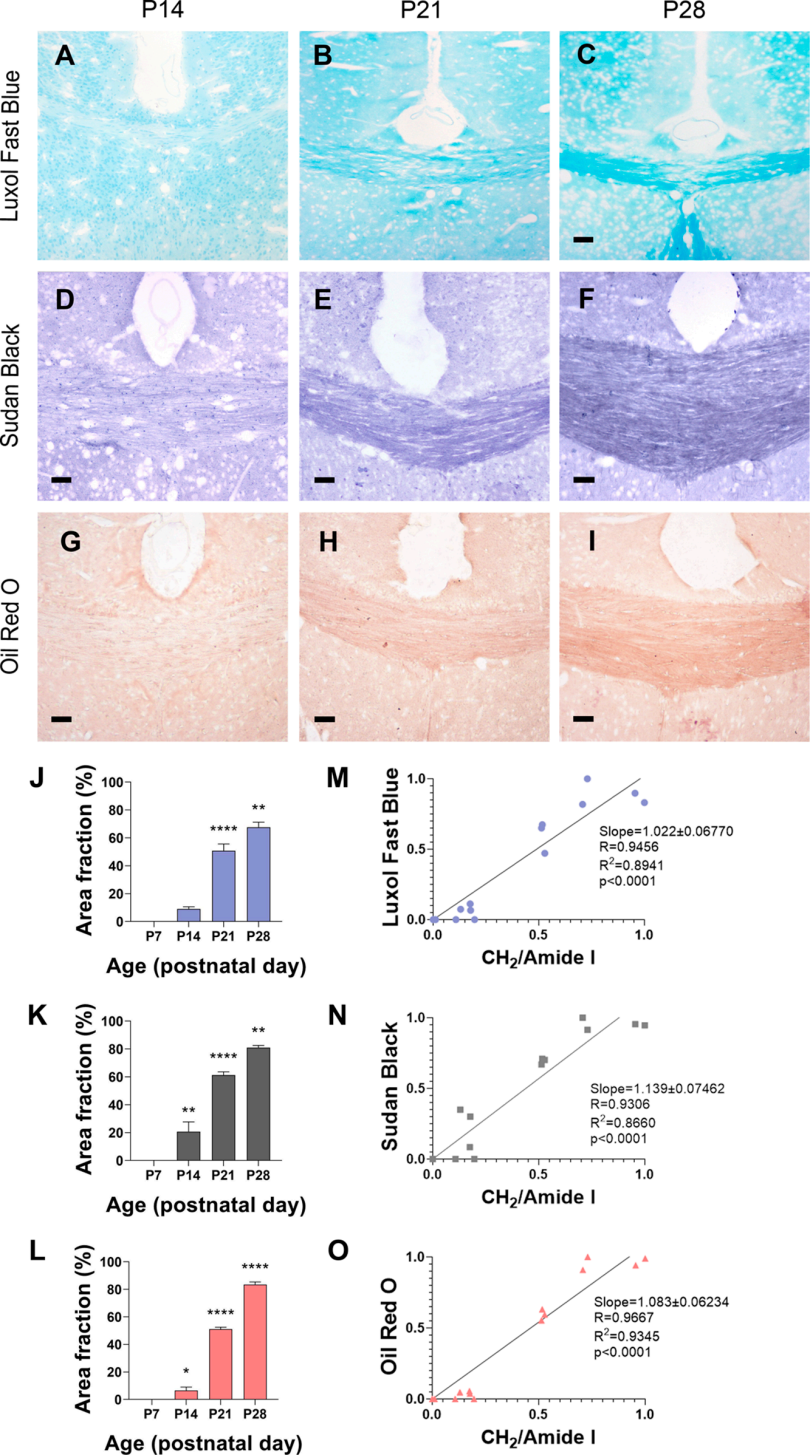
Histochemical lipid staining of brain WM in WT mice during postnatal development. (A–I) Representative microphotographs of (A–C) LFB, (D–F) SB, and (G–I) ORO staining of the CC of WT mice from postnatal day (P14) to P28. (J–L) Quantification of the stained area (%) of (J) LFB, (K) SB, and (L) ORO staining in the CC of WT mice. Statistical treatment was performed with a one-way ANOVA test (time effect *p* < 0.0001) and Tukey’s posthoc comparison (****p < 0.0001, **p < 0.01, and *p < 0.05 compared to the previous time point). Data are represented as median ± SEM (M–O) Correlation between the normalized CH_2_/amide I ratio of the CC and (M) LFB, (N) SB, and (O) ORO staining. See Materials and Methods for further details about the obtention of normalized values. Scale bar: 20 μm.

**Figure 6:**
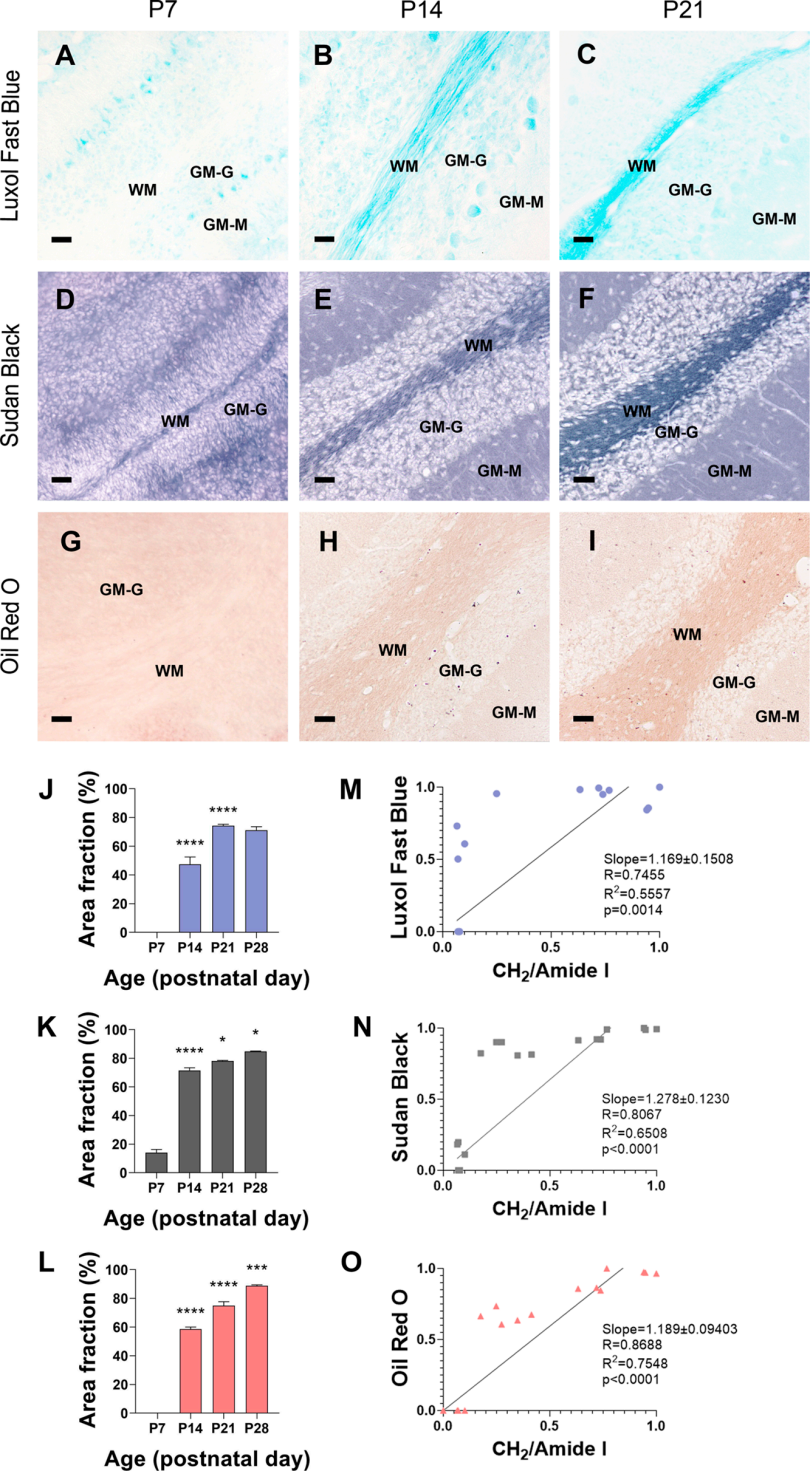
Histochemical lipid staining of cerebellum WM in WT mice during postnatal development. (A–I) Representative microphotographs of (A–C) LFB, (D–F) SB, and (G–I) ORO staining of the arbor vitae of WT mice from postnatal day 7 (P7) to P21. (J–L) Quantification of (J) LFB, (K) SB, and (L) ORO staining in WT. Statistical treatment was performed with the one-way ANOVA test (time effect *p* < 0.0001) and Tukey’s posthoc comparison (****p < 0.0001, ***p < 0.001, and *p < 0.05 compared to the previous time point). Data are represented as median ± SEM (M–O) Correlation between the CH_2_/amide I ratio and (M) LFB, (N) SB, and (O) Oil Red, O staining. See Materials and Methods for further detail about the obtention of normalized values. Scale bar = 20 μm. WM = white matter, GM-M = molecular layer of gray matter, and GM-G = granular layer of gray matter.

First, we compared the WM brain lipid composition with different lipid stains, such as LFB, SB, and ORO (Figures 5 and 6). For all lipid stains, no specific staining was detected in the WM at P7. Staining was detected at P14 and increased rapidly and significantly at P21 and P28 (Figure 5A–L). To determine whether staining reflected trustworthy changes in lipid brain composition, μFTIR values were correlated to the area of positive staining for LFB, SB, and ORO. The CC results showed a high linear correlation between lipid content detected by μFTIR and the intensity of lipid staining (Figure 5M,O). All staining results presented high linear correlations (*R*^2^ > 0.850).

In the cerebellar WM (arbor vitae), we found positive staining at P7 in the SB but not in the LFB and ORO (Figure 6A,D,G). All stains showed a steep increase at P14 and progressed to a slower path until P28 (Figure 6A–L). Quantification of the percentage of stained area revealed progressive significant increases in all staining from P14 to P21, and later to P28, except for LFB, which achieved maximal staining at P21 (Figure 6J–L). Results in the arbor vitae reflected that the myelination process starts earlier in the cerebellum than in the brain and also showed high linear correlations between μFTIR data and lipid staining in SB and the ORO and moderate linear correlations with the LFB (Figure 6M,O).

### 2.4 Modifications in Protein Structure Composition Are Related to the Increase of Myelination Proteins in WM

The general decrease found in the β-sheet/α-helix protein ratio of the WM of both the brain and cerebellum (Figure 4) is coincident with the time points of myelination. Then, we assessed whether changes in the proportion of α-helix and β-sheet proteins in cerebral and cerebellar WM reflect the new synthesis of myelin-associated proteins during primary myelination. As a reference, we performed two immunohistochemical staining against two proteins abundant in myelinated areas: the MBP and the MOG (Figure 6). These two proteins were chosen for their different compositions in secondary structures since MBP is a protein-rich in α-helices [Uniprotkb: P04370], whereas MOG is rich in β-sheets [Uniprotkb: Q61885].

**Figure 7:**
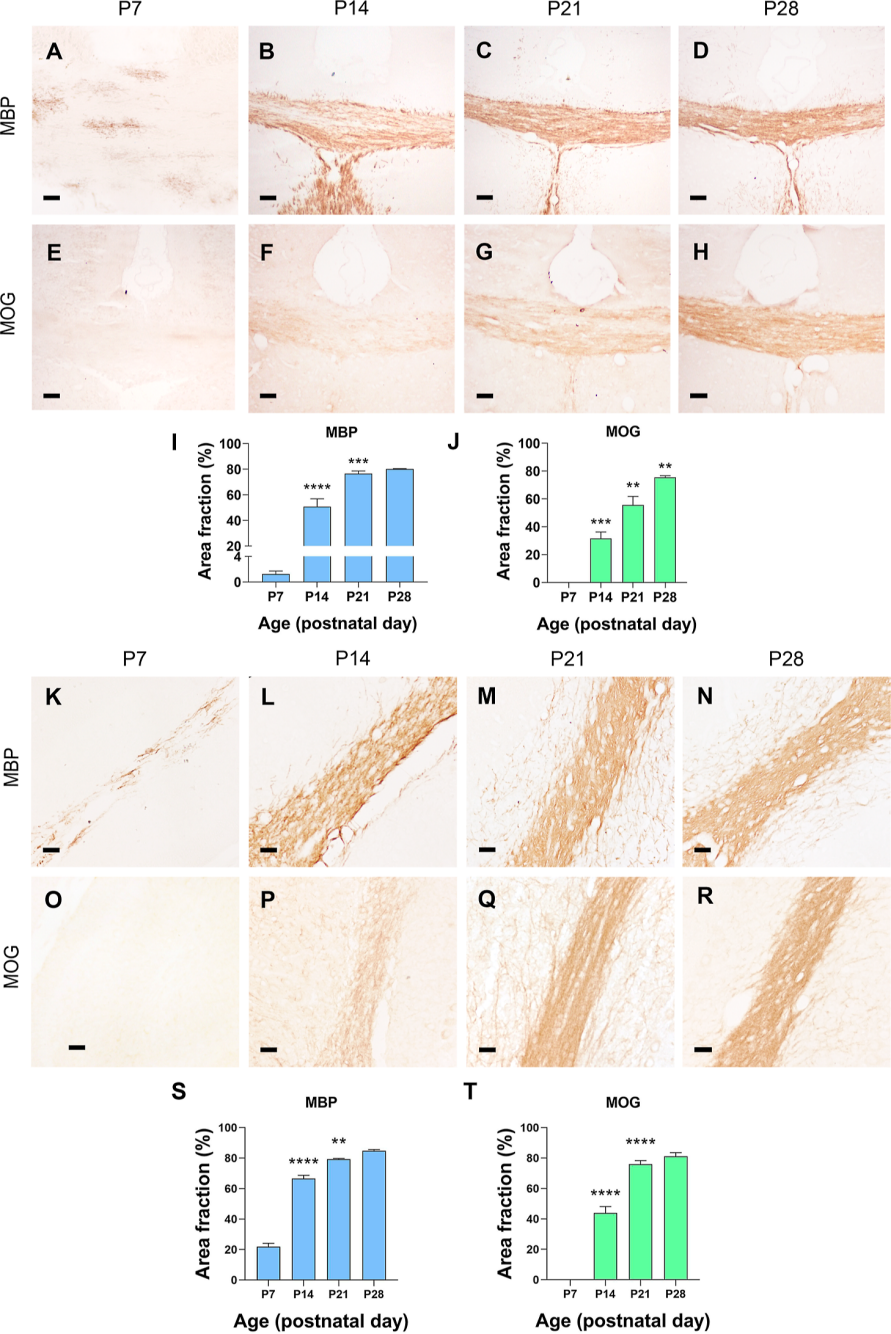
Immunostaining of myelin-associated proteins MBP and MOG in the brain and cerebellar WM of WT mice during postnatal development. (A–H,K–R) Representative microphotographs of the immunohistochemical staining of MBP and MOG in the CC (A–H) and arbor vitae (M–T) of WT mice. (I,J,S,T) Quantification of the percentage of area immunostained by MBP and MOG in CC and arbor vitae. Statistical treatment of a one-way ANOVA test (time effect *p* < 0.0001 for the brain and cerebellum) and Tukey’s posthoc comparison (****p < 0.0001, ***p < 0.001, **p < 0.01, *p < 0.05 respect to the previous time point). Data are represented as median ± SEM. Scale bar = 20 μm.

In the CC, MBP began its expression at P7, where oligodendrocytes were marked with extensions that began to myelinate (Figure 6A). At P14, myelinated fibers were observed throughout the CC, and at P21 and P28, the entire CC was stained, showing complete myelination (Figure 7B–D). Surprisingly, MOG showed a later expression onset than MBP since it started to be expressed at P14 (Figure 7E,F,J). From P14 onward, MOG expression increased gradually until P28 (Figure 7F–H,J). In the cerebellum, we observed a very similar dynamic for MBP and MOG expression in the WM (Figure 6L–U). For MBP and MOG proteins, expression started at either P7 or P14, respectively, as was also detected in CC (Figure 7L,Q).

## 3 Discussion

The present work describes in detail changes in lipids and proteins during CNS development in mice from birth through postnatal stages at the very beginning of life. In addition, we correlated our IR data on biochemical changes in WM with the results of the histochemical and immunohistochemical techniques commonly used to study myelination. In this work, we fixed our samples for preservation purposes and to be able to directly compare our samples in the histochemical study. The effect of fixation in the IR spectra has shown contradictory results in several works.^28–31^ Our results in the GM and WM areas of P28, the age closer to adult mice, are consistent with those shown in mice, rats, and humans independently of tissue processing.^24,32–34^ In our study, the main limitations of sample fixation were the inability to analyze phosphate and carbohydrate regions due to the use of phosphate buffer (PB) and sugar-based cryoprotectants.

During postnatal CNS development, the main differences in composition were detected in the WM. Changes in brain composition during postnatal development are mainly concentrated in lipids, as observed and measured in the IR spectra (Figures 1, 2, S2, and S3), and these modifications are stronger in the WM than in the GM, as expected. The study of GM showed that at P0, the cerebral cortex contained lower amounts of lipids and fewer unsaturated olefinic functional groups than the brain WM (Figure 3). These differences between GM and WM were reduced or lost at later time points (P7, P14) due to the total increase in lipids containing both functional groups (Figure 2). In our study, the stable composition of GM from P7 onward represented a reliable internal control of myelination. Changes in brain GM lipid content and composition at early postnatal stages, from P0 to P7, had been similarly observed when comparing the hippocampus of P6 to P30 rats^27^ and could be explained by the peak of dendrite and axon extension as well as synaptogenesis in cortical areas.^35,36^ Newly formed synaptic connections increase the number of cell membranes, which explains the increase in the CH_2_/amide I ratio and carbonyl and unsaturated olefinic functional groups. In addition, the β-sheet/α-helix ratio raised at P7, as similarly observed in the rat hippocampus,^27^ indicating that β-sheet proteins could increase proportionally during this process. In the cerebellum, an increase in the number of unsaturated olefinic groups was first detected in the molecular layer and then in the granular layer. These specific changes could be related to the migration of granular neurons from the outer to inner cortical areas at these stages.^37^ Detection of those modifications opens the door to the study of changes in GM composition and their relation to hallmarks of CNS formation, such as neuronal migration and/or synaptogenesis.

In WM, primary myelination is represented by an abrupt lipid increase, a process that occurs during postnatal CNS development in mice, rats, and rabbits and in perinatal human stages.^7,16,38^ Accordingly, our data showed a significant increase in lipids of the cerebellar arbor vitae at P14 and later at P21 in the brain CC, which progressed at later time points in both areas (Figures 2 and 3). These results reflect the cauda-rostral directionality of myelination in the CNS, as observed in mice, rats, and humans.^38^ Our results also reinforce a threshold previously proposed to distinguish WM-GM of 1.5 or 1.05 for the CH_2_/amide I ratio, based on data from GM and WM areas in adult mice and the human brain.^19,23^ The WM-GM myelination threshold of 1.05, proposed by Bonda et al.,^24^ specifically distinguishes between lack of or emergent myelin synthesis and partially myelinated WM. We and others have reported myelin formation at P7 in the cerebellum and P14 in the brain, according to the histological detection of lipids, MBP, and MOG proteins,^16,39^ although the increase in lipids at P14 and P21 detected by μFTIR did not reach full myelination. The threshold of 1.5, proposed by Sanchez-Molina et al.,^19^ was achieved in the cerebellar arbor vitae at P14 and in the CC at P21, time points that coincided with significant increases in the CH_2_/amide I ratio, and that were only surpassed at later stages. Based on these observations and our μFTIR data, we propose that the CH_2_/amide I threshold of 1.5 distinguishes well between incomplete and completely myelinated WM since partially myelinated WM is found close to 1.5 but fully myelinated WM is above this threshold. The reliability of this threshold is also reinforced by the fact that the lipid–protein ratio in myelin is maintained throughout life, as reported by others, meaning that its lipid–protein ratio is constant;^5^ therefore, the increase in lipids observed in the quantification of the CH_2_/amide I ratio WM reflects myelination. Beyond the myelination thresholds, primary myelination progresses further at P14 in the cerebellum and P21 in the CC. Because the CH_2_/amide I ratio at P28 approaches that described for CC WM in the adult brain, approximately 2.0,^19^ we could say that in this study, we characterized the full primary myelination process. Nevertheless, it should be kept in mind that myelination of WM and other brain areas progresses further in the postnatal brain,^38,40^ and according to some studies, whole-brain myelination is not completed at most until 3 months in mice/rats^8^ or until adolescence in humans.^41^

Analysis of functional groups using μFTIR revealed the biochemical tissue composition in postnatal WM areas more specifically (Figure 3). Unsaturated olefinic functional groups are found in higher amounts in polyunsaturated long-chain fatty acids, while carbonyl functional groups are found in lipids containing ester groups, and they can be quantified through μFTIR spectra.^34,42,43^ In our work, both olefinic unsaturated and carbonyl contents proportionally decreased compared to the total lipid content, while concomitantly, the global WM lipid amount increased. Our results suggest that the WM composition is poorer in lipids containing unsaturated and carbonyl functional groups during myelin maturation. This interpretation is supported by the strong negative linear correlations obtained for carbonyls in relation to the CH_2_/amide I ratio in the brain CC and cerebellar WM. Our results agree with a recent report describing a shift from phospholipids to monounsaturated fatty acids when comparing P15 to P40 myelin-enriched mouse brain tissues.^9^ Similarly, previous studies found an increased content of polyunsaturated fatty acids in the forebrain of the human brain during development, whereas myelin-specific lipids contained more polysaturated long-chain fatty acids and monounsaturated fatty acids, specifically oleic acid.^14^ In agreement with the tendency described, in both adult human and mouse brain tissue, WM contained a lower content of unsaturated olefinic and carbonyl bonds compared to GM.^19,20^ These results can be explained by the elevated content of monounsaturated fatty acids in WM and polyunsaturated fatty acids in GM.^20^ The changes observed in the spectra during WM maturation could be because of myelin accumulation, which has a different molar ratio of cholesterol, phospholipids, and glycosphingolipids (40:40:20) than cell membranes (25:65:10).^42,43^ Lipids found most abundantly in myelin are cholesterol, galactosylceramides, ethanolamine plasmalogen, and sphingomyelin.^9,42–45^ Carbonyl and unsaturated olefinic functional groups are absent in cholesterol, sphingomyelin, and long chains of monounsaturated fatty acids but are present in some phospholipids.^46^

Histochemical and immunohistochemical techniques, including lipid staining, have been extensively used to describe changes in WM in physiological and pathological contexts, such as demyelination/remyelination.^16,47–49^ Lipid staining used in this study is one of the most popular methods for characterizing myelin^49,50^ and shows different properties. ORO stains hydrophobic or neutral lipids, such as fats and cholesterol esters of unsaturated fatty acids, and SB shows a similar pattern to ORO, in which less hydrophobic lipids, such as phospholipids and sphingolipids, are also stained.^47,51^ LFB is usually referred to as phospholipid staining, although this association is unclear.^47,52^ The CH_2_/amide I ratio was highly correlated with the intensity of three different lipid histological staining results in both the CC and cerebellar arbor vitae (Figures 5 and 6). As mentioned above, the three-lipid staining began to stain some early fibers at a time point before the rise in the CH_2_/amide I ratio in both the brain and cerebellum. ORO, SB, and LFB staining showed a strong linear correlation with lipid increase in the CC, and only LFB showed a moderate linear correlation in the cerebellum.

Immunohistochemical staining of myelin proteins is widely used to track the degree of myelination. The most abundant proteins in myelin are PLP (38%), MBP (30%), 2′,3′-cyclic nucleotide 3′-phosphodiesterase (5%), and MOG (1%).^52^ Thus, MBP is one of the most commonly used proteins to determine myelination and demyelination in CNS tissues. In our study, MBP was detected in a few mature oligodendrocytes in the CC and whole fibers in the cerebellar WM before ORO, LFB, or SB (Figure 7). MBP is found in the outer lamellar sheet of myelin and supports myelin compacting and adhesion.^38,53^ Its appearance has been described before myelin formation in the extracellular membranes of mature oligodendrocytes.^54^ Because MBP onset is related to lipid staining and the CH_2_/amide I ratio increases, the use of MBP to determine the degree of myelination should be carefully evaluated, especially at early stages, as MBP expression does not reflect a full and compact myelin lipid envelope around axons. In contrast, in our study, MOG appeared later than MBP because its expression has been described in myelinating rather than all mature oligodendrocytes.^55,56^

Myelin compaction is supported by two major myelin-associated proteins: MBP and PLP. As mentioned above, MBP expression does not accurately reflect myelination; however, myelin bundle formation is associated with protein post-transduction modifications and changes in their spatial organization.^56^ Examination of the secondary structure by our μFTIR data showed a decrease in the intramolecular β-sheet/α-helix ratio (Figure 4), which could be explained by the increase in the proportion of α-helix protein domains rather than intramolecular β-sheets in proteins found in WM during primary myelination. These data agree with those of previous studies reporting a lower intramolecular β-sheet/α-helix ratio in WM compared to GM in both human and mouse brain tissue.^19^ Furthermore, a shift from the α-helix to the intramolecular β-sheet has been detected by μFTIR in the peri-infarct zone of rat brain ischemic lesions or in the CC after traumatic brain injury, coinciding, in the first study, with myelin loss detected by electron microscopy.^57,58^ Changes in protein structure have been related to different functions, and indeed, its alteration has been associated with demyelination.^57–59^ This early evidence suggests that properly compacted and functional myelin favored the α-helix conformation of the main compacting proteins MBP and PLP. In support of this data, PLP, the most abundant protein in CNS myelin, contains several domains that adopt an α-helical structure in lipid bilayers.^60^ MBP has specific domains with α-helix and β-sheet conformational states, but the α-helix domain increases with lipid interactions, especially more with saturated than unsaturated fatty acids.^54,61–64^ Therefore, in this study, we suggest that myelin bundle formation and its alterations could be detected by measuring intramolecular β-sheet/α-helix ratios in WM. In some contexts, using this approach to evaluate myelination could be advantageous over other expensive and time-consuming techniques, such as transmission electron microscopy.

## 4 Conclusions

In this work, we described the changes in lipid and protein compositions during postnatal CNS development in the brain and cerebellar GM and WM using μFTIR in detail. Our data provide new insights into the most significant changes related to primary myelination in WM, consisting of an increase in lipids containing low olefinic unsaturation and carbonyls. For the first time, we describe how a proper degree of myelination is related to a lower intramolecular β-sheet/α-helix ratio in WM than in GM. Our data showed that primary myelination may be better studied with routine lipid histochemical staining than with immunohistochemical staining against some myelin-associated proteins, such as MBP. These data provide a reference for further studies determining alterations in experimental animals with neurodevelopmental diseases and constitute a basis for studying brain composition during human perinatal development using μFTIR. In this study, we also showed how the use of IR microscopy can easily provide complete data on the lipid and protein content of specific GM and WM areas and the degree of myelination with little biological material.

## 5 Materials and Methods

### 5.1 Animals

C57BL/6 mouse pups P0 to P28 of both sexes were used in this study. Pups from P0 to P21 were bred and maintained with their mothers until they were euthanized. After weaning at P21, P28 animals were housed and bred in the Animal House of the Institute of Neurosciences of the Universitat Autònoma de Barcelona under a 12 h light/dark cycle and maintained at a constant temperature (24 ± 2 °C) with food and water ad libitum. All experimental animal studies were conducted according to Spanish regulations (Ley 32/2007, Real Decreto 1201/2005, Ley 9/2003, and Real Decreto 178/2004) in agreement with European Union directives (86/609/CEE, 91/628/CEE, and 92/65/CEE) and were approved by the Ethical Committee of the Autonomous University of Barcelona. Every effort was made to minimize the number of animals used to produce reliable scientific data, as well as animal suffering and pain. Animals were grouped according to age as follows: postnatal day 0 (P0), P7, P14, P21, and P28. Each experimental group consisted of 4–5 animals.

### 5.2 Tissue Sample Obtention of Cryostat Sections for Histological and μFTIR Analysis

Mouse pups were anesthetized intraperitoneally (ip) with a solution of ketamine (80 mg/kg) and xylazine (20 mg/kg) at a dose of 0.015 mL/g and intracardially perfused with 4% paraformaldehyde in 0.1 M PB (pH 7.4) for 5 or 10 min (P0 to P7 or P14 to P28, respectively). The brain was immediately removed and postfixed by immersion in the same fixative solution for 4 h at 4 °C. Samples were then washed with PB and cryoprotected for 48 h with a 30% sucrose solution in PB. Then, the cerebellum was separated from the brain before being frozen using methylbutane (320404, Sigma-Aldrich) and chilled in dry ice. The samples were stored at −80 °C until use.

For either μFTIR analysis or histology, 9 μm-thick frozen coronal brain and sagittal cerebellar sections were cut using a Leica CM3050 cryostat. Sections for μFTIR analysis were mounted onto polished calcium fluoride (CaF_2_) optical windows (CAFP20-1; Crystran, UK). To minimize the water contribution, sections on the CaF_2_ slides were air-dried at room temperature (RT) and stored in a vacuum protected from light until use. Cryostat sections for histology were mounted on Flex IHC slides (Dako, Glostrup, Denmark). Three consecutive coronal brain slices (P0 #26,^65^ 3.51 mm bregma^66^) and medial sagittal cerebellum slices were obtained for each animal. Once collected, the slides were stored at −20 °C until use.

### 5.3 Tissue Sample Obtention of Paraffin Sections for Histological Analysis

Mouse pups from P7 to P28 were intracardially perfused, as described above. Brains were dissected and postfixed by immersion in the same fixative solution for 24 h at 4 °C. After three washes in PB for 30 min each, the brain and cerebellum were separated, and the samples were progressively dehydrated in 50, 70, and 95° and absolute ethanol, cleared in xylene, and embedded in paraffin blocks. 9 μm-thick coronal brain sections and sagittal cerebellar sections were cut using a Leitz 1512 rotatory microtome and collected on gelatinized slides. Coronal brain slices ranged from 2.79 to 5.07 mm in P7 samples (P6 #12 to #31)^65^ and from bregma 1.10 to −2.64 mm in P14, P21, and P28 samples.^66^ The parasagittal cerebellar slices ranged from central to lateral 0.04 mm.

### 5.4 LFB Staining

Myelin staining was performed, as described by Petkovic et al.^67^ Coronal (in brain samples) or sagittal (in cerebellum samples) paraffin sections were deparaffined, hydrated, and incubated in LFB solution (0.1 g of LFB/100 mL of 95% ethanol with 10.5 mL of 10% CH_3_COOH) for 3 h at 60 °C. The excess was washed with 95% ethanol and distilled water, and the sections were differentiated using 0.05% Li_2_CO_3_ solution, followed by 70% ethanol. Finally, the sections were dehydrated in alcohol, cleared in xylene, and covered with a DPX mounting medium.

### 5.5 SB Staining

SB staining was performed according to the method described by Ineichen et al.^68^ Coronal brain and sagittal cerebellum cryostat sections were air-dried and washed with 0.05 M Tris buffer. The slices were then incubated in a freshly made and filtered SB solution (1% SB in ethylene glycol) for 20 min at RT. After washing off excess SB solution with 0.05 M Tris buffer, slides were cover-slipped with PermaFluor aqueous mounting medium (TA-030-FM, Thermo Fisher Scientific).

### 5.6 ORO Staining

For ORO staining, coronal brain and sagittal cerebellum cryostat sections were incubated in freshly made and filtered ORO solution (ORO stock solution: 2.5 g/400 mL 99% isopropyl alcohol; ORO working solution: 1.5 stock solution/1 distilled water) for 2 h at RT.^43^ Excess was washed with tap water, and the slides were cover-slipped with PermaFluor aqueous mounting medium (TA-030-FM, Thermo Fisher Scientific).

### 5.7 Single Immunohistochemistry

Paraffin-embedded coronal brain and sagittal cerebellar sections were immunostained for the visualization of myelin (MBP and MOG). Sections were washed twice in 0.05 M Tris-buffered saline (TBS, pH 7.4) for 5 min, blocked for endogenous peroxidase by incubating 2% H_2_O_2_ in 70% methanol for 5 min, and washed again in 0.05 M TBS containing 0.1% Triton X-100 (TBS-0.1%T). Sections were then blocked for nonspecific staining by incubating in a blocking buffer solution (BB, containing 10% fetal bovine serum and 0.3% bovine serum albumin in TBS-0.1%T) for 1 h at RT. Next, the sections were incubated with the primary antibody (rat anti-MBP -ab7349 Abcam-, 1:500; rabbit anti-MOG -859901 Biolegend-, 1:50) overnight at 4 followed by 1 h at 37 °C. Sections were rinsed three times in TBS-0.1%T and incubated with an anti-rat (BA-4001 Vector Laboratories) or anti-rabbit (BA-1100 Vector Laboratories) secondary antibody diluted in BB (1:500) for 1 h at RT. After washing with TBS-T, the sections were incubated with streptavidin-peroxidase (1:500 in BB, SA-5004 Vector Laboratories) for 1 h at RT and then washed twice in TBS and subsequently with TB (0.05 M Trizma base, pH 7.4). The reaction was visualized using a DAB kit (SK-4100; Vector Laboratories, Inc., Burlingame, CA, USA), following the manufacturer’s instructions. Finally, the sections were dehydrated in alcohol, cleared in xylene, and cover-slipped in the DPX mounting medium. The sections were observed using a bright-field Nikon Eclipse 80i microscope.

### 5.8 Densitometric Analysis of Histochemical and Immunohistochemical Staining

For immunostaining analysis, images of a minimum of three slices per animal were captured at 20× magnification with a DXM 1200F Nikon digital camera mounted on a bright-field Nikon Eclipse 80i microscope and using ACT-1 2.20 software (Nikon Corporation). For each brain slice, three photographs were taken corresponding to the central and lateral regions of the CC and three photographs corresponding to the cerebral cortex. For cerebellum slices, three photographs were taken corresponding to regions of the arbor vitae and the two layers of its GM: the molecular layer and the granular layer.

Densitometric analysis for each photograph was performed using Analysis software. The percentage of the area occupied by immunolabeling (% area) was recorded for each photograph.

### 5.9 μFTIR Data Acquisition

μFTIR based on synchrotron radiation was performed at the MIRAS beamline of the ALBA Synchrotron light source (Catalonia, Spain).^69^ The measurements were performed as described by Sánchez-Molina et al.^19^ In brief, a Hyperion 3000 microscope with a 36× objective coupled to a Vertex 70 spectrometer (Bruker, Billerica, MA, USA) was used. Spectra were collected in transmission mode at 4 cm^–1^ resolution, 10 × 10 μm aperture, 128 scans in brain samples, and 64 scans in cerebellum samples using the Opus 7.5 software (Bruker, Billerica, MA, USA). Measurements ranged from 4000 to 600 cm^–1^ wavenumbers, and zero filling was performed with a fast Fourier transform, obtaining one point every 2 cm^–1^. Background spectra were collected every 10 min in each CaF_2_ window from a clean area. For each region of interest, approximately 50 spectra with a step size of 30 μm × 30 μm were acquired. To represent regional differences with high spatial resolution in the tissue, maps of 100 spectra with a step size of 6 × 6 μm were generated, producing a final map of 60 × 60 μm in one representative sample of each species containing GM (cortex in the brain and molecular and granular layers in the cerebellum) and WM areas (CC in the brain and arbor vitae in the cerebellum).

### 5.10 μFTIR Spectrum Analysis

Unscrambler X software (CAMO Software, Oslo, Norway) was used for data processing. The second derivative of the spectra was calculated using the Savitzky–Golay algorithm with an 11-point smoothing filter and a polynomial order of 3 to enhance the narrow bands and eliminate the baseline contribution. With the data already processed, we analyzed the absorbances of the following bands:^70^ 3012 cm^–1^ (C=CH, unsaturated olefinic group), 2852 cm^–1^ (CH_2_ symmetric stretching vibrations), 2921 cm^–1^ (CH_2_ asymmetric stretching vibrations), 1743 cm^–1^ (C=O, carbonyl group), 1656 cm^–1^ (α-helix secondary protein structure), and 1637 cm^–1^ (β-sheet secondary protein structure). The values mentioned above were obtained by calculating the average of the band at 2 cm^–1^ before and 2 cm^–1^ after from the secondary derivative of each spectrum. CH_2_ is a functional group, especially present in lipids; therefore, it was used to normalize the values using it as the total lipid value. To study the secondary structure of the proteins, the α/β ratio was calculated by taking the absorption values of the α structures at 1656 cm^–1^ and the intramolecular β structures at 1637 cm^–1^ of the second derivative spectra (d^2^*A*_1637_/d^2^*A*_1656_). The sum of both bands was used as the total protein value.

### 5.11 Statistical Analysis

Statistical analyses were performed using the GraphPad Prism software (version 8.0; GraphPad Software, San Diego, California, USA). All experimental results are expressed as the mean value ± SEM. To study differences in age and region, a two-way ANOVA with Tukey’s post-hoc test was used to compare postnatal days and studied areas. A one-way ANOVA was performed to analyze lipid staining and immunohistochemistry. Statistical significance was set at *p* < 0.05.

To study whether the increase or decrease in lipids was related to changes observed in functional olefinic unsaturated or carbonyl groups or increased histochemical lipid staining, either absorbance ratios or the percentage of stained area were normalized between 0 and 1 by considering the maximal value equivalent to 1, as performed in Benseny-Cases et al.^25^ Correlations were determined using Pearson’s coefficient (R > 0.80 or < −0.80, strong correlation; R > 0.60 or < −0.60, moderate correlation), coefficient of determination (R^2^ correlation <0.65, poor correlation), and Student’s *t*-test (*p* < 0.05). A good linear correlation was considered when the slope was close to 1 (good correlation >0.8500 or < −0.8500).

## Supporting Information Available

μFTIR average absorbance spectra; total lipid and protein content in brain GM and WM; and total lipid and protein content in the cerebellum GM and WM (PDF)
