# Supplementary material for: Roadmap for Postnatal Brain Maturation: Changes in Gray and White Matter Composition during Development Measured by Fourier Transformed Infrared Microspectroscopy
Source: ACS Chem Neurosci. 2023 Aug 4;14(17):3088–102. doi: 10.1021/acschemneuro.3c00237 (PMC10485886; doi:10.1021/acschemneuro.3c00237)
Supplement: Supplementary file 1 — cn3c00237_si_001.pdf [file cn3c00237_si_001.pdf]

A Roadmap for Postnatal Brain Maturation: Changes in Grey and White Matter Composition during Development Measured by Fourier Transformed Infrared Microspectroscopy.

Marta Peris[1], Núria Benseny-Cases[2]*, Gemma Manich[3], Oriana Zerpa[1], Beatriz Almolda[1], Àlex Perálvarez-Marín[2], Berta González[1] and Bernardo Castellano[1]*

1) Department of Cell Biology, Physiology and Immunology, Institute of Neuroscience. Universitat Autònoma de Barcelona, Bellaterra, 08193 Barcelona, Spain.
2) Biophysics Unit. Department of Biochemistry and Molecular Biology. Universitat Autònoma de Barcelona, Bellaterra, 08193 Barcelona, Spain.
3) Department of Morphological Sciences, Universitat Autònoma de Barcelona, Bellaterra, 08193 Barcelona, Spain.

*equally contributed to the study

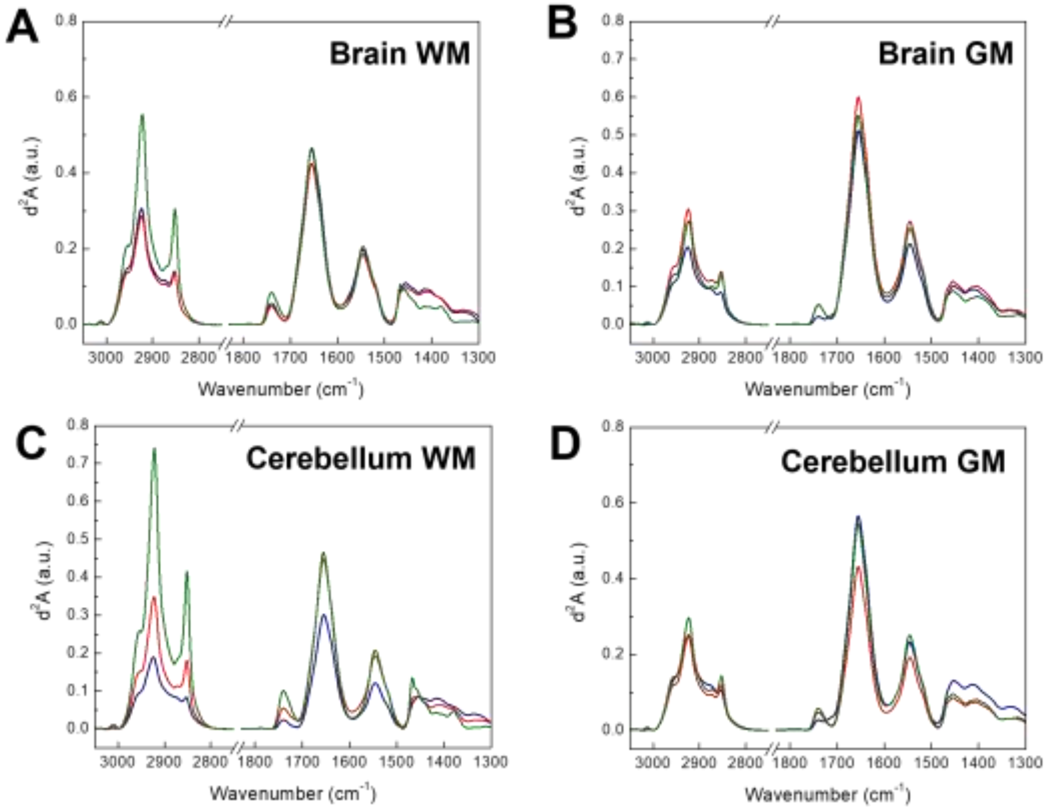

Supplementary Figure S1. μFTIR absorbance spectra of brain and cerebellum WM and GM during postnatal development. Average spectrum of white and grey matter areas of the mouse brain (A-B) and the cerebellum (C-D) at P0 (blue lines), P14 (red lines) and P28 (green lines) after baseline correction. Peaks corresponding to functional groups are illustrated in groups according their absorbance (in arbitrary units) of the following bands (wavenumber in cm^{-1}): 3012 cm^{-1} (C=CH, unsaturated olefinic group), 2921 cm^{-1} (CH_2 asymmetric stretching vibrations), 1743 cm^{-1} (C=O, carbonyl group), 1656 cm^{-1} (α-helix secondary protein structure), 1637 cm^{-1} (β-sheet secondary protein structure).

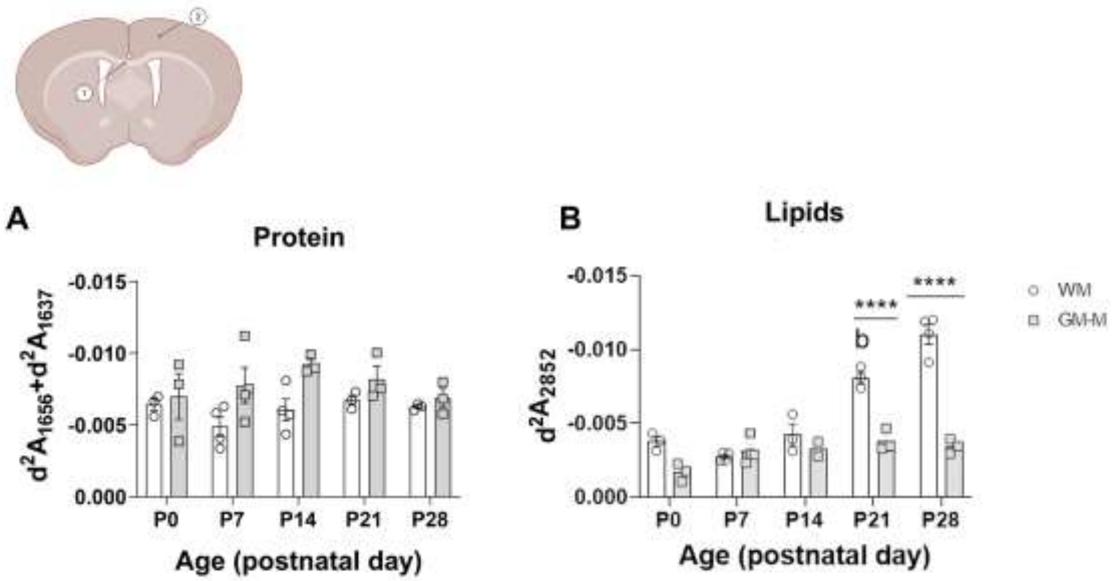

Supplementary Figure S2. Total lipid and protein content in brain white matter (WM) and grey matter (GM) of WT mice during postnatal development. (A) Representation of the second derivate absorbances of protein (α+β) content from postnatal day 0 (P0) to P28. **(B)** Representation of the second derivate absorbances of symmetric CH_2 (A_{2852}) as a demonstration of total lipid (CH_2) from postnatal day 0 (P0) to P28. Data are represented as median± SEM. Statistical treatment was performed with two-way ANOVA test (time effect ****p<0.0001 in lipids A_{2852}, regional effect ****p<0.0001 in lipids A_{2921} and A_{2852}; time effect ns in proteins; regional effect *p<0.05 in proteins), and Tukey's post-hoc comparisons (*p<0.05, ***p<0.001, ****p<0.0001 WM compared to GM; "b" p<0.001 compared to the previous time-point). Top left a drawing where the studied areas are indicated: 1-corpus callosum, 2-cortex; created with Biorender.com.

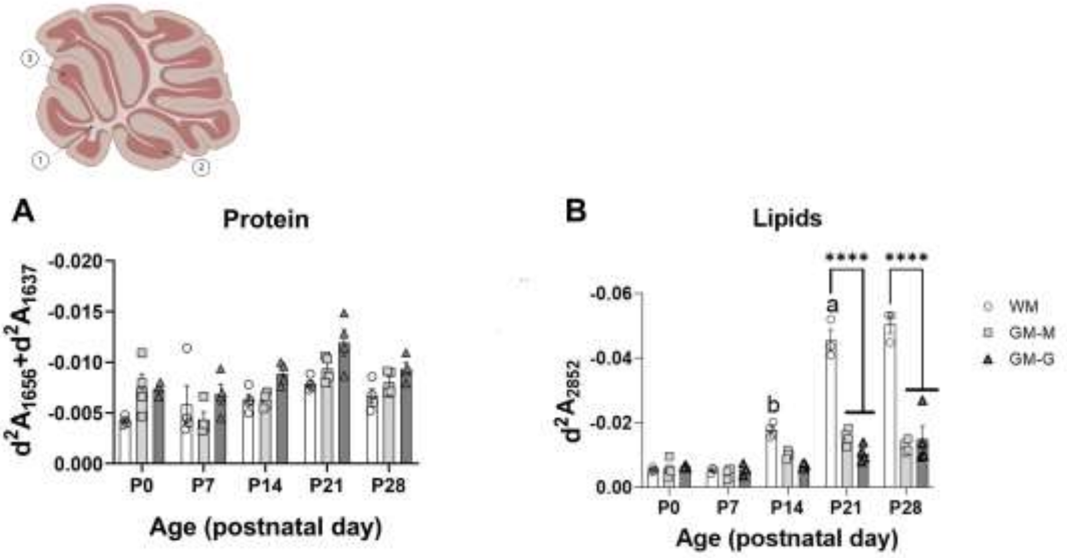

Supplementary Figure S3. Total lipid and protein content in cerebellum white matter (WM) and molecular (GM-M) and granular (GM-G) layers of cerebellar grey matter of WT mice during postnatal development. (A) Representation of the second derivate absorbances of protein (α+β) content from postnatal day 0 (P0) to P28. **(B)** Representation of the second derivate absorbances of symmetric CH_2 (A_{2852}) as a demonstration of total lipid (CH_2) from postnatal day 0 (P0) to P28. Data are represented as median± SEM. Statistical treatment was performed with two-way ANOVA test (time effect ****p<0.0001 in lipids A_{2852}, regional effect ****p<0.0001 in lipids A_{2852}; time effect ns in proteins; regional effect *p<0.05 in proteins), and Tukey's post-hoc comparisons (****p<0.0001 WM compared to GM; "a" p<0.0001; "b" p<0.001 compared to the previous time-point). Top left a drawing where the studied areas are indicated: 1-arbor vitae, 2-granular layer, 3-molecular layer; created by Biorender.com.
